# Comprehensive analysis of genetic associations and single-cell expression profiles reveals potential links between migraine and multiple diseases: a phenome-wide association study

**DOI:** 10.3389/fneur.2024.1301208

**Published:** 2024-02-07

**Authors:** Di Ouyang, Chunying Huang, Huihua Liu, Weiming Xie, Chengsheng Chen, Ben Su, Lizhong Guo

**Affiliations:** ^1^Nanjing University of Chinese Medicine, Nanjing, China; ^2^Traditional Chinese Medicine Hospital of Yulin, Yulin, China; ^3^Guangxi Medical University, Nanning, China; ^4^Hubei University of Chinese Medicine, Wuhan, China; ^5^Shanghai University of Traditional Chinese Medicine, Shanghai, China

**Keywords:** migraine, microglia, phenome-wide association study, single-cell RNA sequencing, genetic association study, susceptibility gen

## Abstract

Migraine is a common neurological disorder that affects more than one billion people worldwide. Recent genome-wide association studies have identified 123 genetic loci associated with migraine risk. However, the biological mechanisms underlying migraine and its relationships with other complex diseases remain unclear. We performed a phenome-wide association study (PheWAS) using UK Biobank data to investigate associations between migraine and 416 phenotypes. Mendelian randomization was employed using the IVW method. For loci associated with multiple diseases, pleiotropy was tested using MR-Egger. Single-cell RNA sequencing data was analyzed to profile the expression of 73 migraine susceptibility genes across brain cell types. qPCR was used to validate the expression of selected genes in microglia. PheWAS identified 15 disorders significantly associated with migraine, with one association detecting potential pleiotropy. Single-cell analysis revealed elevated expression of seven susceptibility genes (including ZEB2, RUNX1, SLC24A3, ANKDD1B, etc.) in brain glial cells. And qPCR confirmed the upregulation of these genes in LPS-treated microglia. This multimodal analysis provides novel insights into the link between migraine and other diseases. The single-cell profiling suggests the involvement of specific brain cells and molecular pathways. Validation of gene expression in microglia supports their potential role in migraine pathology. Overall, this study uncovers pleiotropic relationships and the biological underpinnings of migraine susceptibility.

## Introduction

Migraine, the third most prevalent disease in the world, is a primary neurological disorder characterized by recurrent episodes of severe headache, often accompanied by debilitating symptoms such as nausea, vomiting and sensitivity to light or sound (1). It is estimated that approximately 1 billion people worldwide suffer from migraines, which severely impact their quality of life and create significant social and economic burdens (2, 3). Although the exact etiology of migraine is unknown, it may result from complex multifactorial influences, including genes, hormones, metabolic status, cervical neuroanatomy and medications (4). Despite its widespread impact, the biological mechanisms underlying migraine are not fully understood, which hinders progress in developing effective treatments and preventive strategies.

Genetic factors are believed to play a critical role in migraine susceptibility (5). Recent advancements in genomics have facilitated large-scale genetic studies, such as genome-wide association studies (GWAS), which have identified numerous genetic loci associated with various diseases, including migraine (6–8). A landmark GWAS by Hautakangas et al. identified 123 genetic loci associated with migraine risk, greatly expanding our understanding of the genetic basis of this debilitating disorder (9). Recent studies have shown that the neurovascular unit plays an important role in monogenic migraines, and genome-wide association studies have identified several susceptibility variants that confer an increased overall risk of migraine (10). However, these genetic findings represent only a small piece of the complex puzzle of migraine etiology, and their functional implications remain largely unexplored. The emergence of phenome-wide association studies (PheWAS) allows for a comprehensive exploration of the relationships between genetic variants and a wide range of phenotypes (11). PheWAS is a complementary approach to GWAS that can uncover previously unknown associations between diseases and potentially reveal common biological pathways and mechanisms (12, 13). Furthermore, by exploiting the known biology behind seemingly disparate phenotypes, PheWAS may provide new insights into the multifaceted nature of migraine.

In addition to genetic associations, examining gene expression at the cellular level can also provide valuable information about disease mechanisms. The recent advent of single-cell RNA sequencing (scRNA-seq) technologies enables precise measurement of gene expression in individual cells, overcoming the limitations of traditional bulk RNA-seq, which provides averaged gene expression across a mixture of cell types (14–16). Applying scRNA-seq to study the brain could reveal cell type-specific expression patterns of migraine-associated genes, which might be crucial for understanding the cellular and molecular underpinnings of this complex disorder. It has been shown that scRNA-seq technology has provided exciting insights into the diversity of human oligodendrocytes and the functional state of human microglia (17–19). As scRNA-seq technology advances, the estimation of genetic variation from scRNA-seq data is becoming more reliable.

In this work, we aim to integrate these advanced genetic and transcriptomic analyses to gain a deeper understanding of migraine. We will first perform a PheWAS to investigate the associations between migraine and a wide range of diseases using data from the UK Biobank. We will then analyze scRNA-seq data to profile the expression of migraine susceptibility genes in different brain cell types. Using multiple approaches, we hope to discover new links between migraine and other diseases, identify potential pleiotropic effects, and elucidate the cellular environment in which migraine-associated genes exert their functions.

## Methods

### Data source

The risk loci for migraine were derived from the work of Hautakangas et al. (9). This study addressed the significant global impact of migraine, affecting over a billion individuals, while its genetic basis largely remained a mystery. Hautakangas et al. conducted a genome-wide association study involving 102,084 migraine cases and 771,257 control subjects, successfully identifying 123 genetic loci, of which 86 were previously unknown. We leveraged this study’s findings to perform a comprehensive PheWAS of migraine. The phenotypic data for the PheWAS were sourced from the UK Biobank, encompassing 416 diseases, including 203 ICD-10 main diagnoses, 213 ICD-10 secondary diagnoses, and 18 types of cancer, with further specifics detailed in Supplementary material S1.

### Data control

For the 123 risk loci, a rigorous data control process was implemented. Initially, variants with a minor allele frequency (MAF) of less than 1% were excluded. Subsequently, linkage disequilibrium (LD) pruning was carried out using LDlink,^1^ retaining variants with the lowest *p*-value within a minimum distance of 10,000 kilobases and an *R*^2^ value less than 0.001. In terms of phenotype selection, given the binary outcome nature of the data, phecodes with fewer than 200 cases were excluded, ensuring a study population with European ancestry.

### MR-PheWAS

We employed Mendelian randomization (MR) methodology to investigate the associations between migraine and various phenotypes, conducting PheWAS. In this study, we utilized the Inverse Variance Weighting (IVW) method to assess these associations. Given the potential correlation among different phenotypes, associations with a *p*-value less than 0.01 were considered meaningful. For those meaningful associations, we applied the MR Egger method to detect potential pleiotropic effects. This analysis was performed using the TwosampleMR package version 0.5.7 in R 4.1.3.

### Single-cell sequencing data analysis

The white matter found within the human central nervous system (CNS) houses a diverse range of non-neuronal glial cells, including oligodendroglia (comprising oligodendrocytes and their precursor cells referred to as OPCs), astrocytes, and microglia. In our research, we utilized single-cell sequencing data derived from the nuclei of white matter samples obtained from deceased, healthy individuals. Specifically, our focus was on three distinct regions within the CNS: the primary motor cortex (Brodmann area 4; BA4), the cerebellum (CB), and the cervical spinal cord (CSC). These samples were collected from two different adult age groups, namely those aged 30–45 years and 60–75 years, encompassing both genders. The dataset comprised a total of 45,528 cells, representing various cell types, including oligodendroglia, neurons, vascular cells, microglia, and astrocytes.^2^

To elucidate the expression patterns of susceptibility genes within these five cell types, we conducted comprehensive analyses. Cell types were identified through principal component analysis, graph clustering of transcriptomic data from individual cells, and *in situ* histochemistry. Data preprocessing steps within CELLxGENE Discover encompassed several critical procedures to ensure the quality and consistency of the analyzed data. Firstly, duplicate cells resulting from independent submissions were removed, retaining only cells marked as primary data. Additionally, cells expressing fewer than 500 genes were excluded. Furthermore, only cells from sequencing assays measuring gene expression and not requiring gene-length normalization were retained. Gene counts were normalized using a rankit method, a variant of quantile normalization. This normalization process transformed read counts across genes into quantiles and mapped them to a standard normal distribution.

Migraine’s 123 risk loci encompassed 73 located within genes. We utilized single-cell expression level analysis to investigate the expression levels of these 73 susceptibility genes across 16 cell types within three distinct regions. We particularly emphasized the expression within microglia. This analysis was conducted using the cellxgene.census 1.5.1 package in R v.4.1.3, integrating information concerning both cell type identification and susceptibility gene expression within individual cells.

### Verification of the expression level of target genes through qPCR

Human microglial cell line 3 (HMC3) cells were cultured in complete medium, composed of DMEM supplemented with 10% FBS and 100 U mL-1 penicillin–streptomycin, at 37°C in a mixed air CO_2_ environment for a period of 24 h. Following this, the cells were segregated into experimental and control groups. The experimental group was treated with Lipopolysaccharides (LPS) at a concentration of 100 ng/mL for 24 h, while the control group remained untreated. RNA isolation was carried out on both groups of cells using an RNA Isolation Kit, according to the manufacturer’s instructions. Subsequently, cDNA synthesis was performed using the First Strand cDNA Synthesis Kit, which served as the template DNA for the real-time qPCR assay. The RT qPCR assay was conducted for four target genes, namely ZEB2, RUNX1, SLC24A3, ANKDD1B, RBM14-RBM4, ASTN2, RABGAP1, and LRCH1, using a SYBR Green qPCR Mix on a real-time detector. Each experiment was repeated thrice to obtain mean values and standard deviation (SD). Statistical analysis was conducted using SPSS Statistics 25, and *p* values were calculated (*p < 0.05, **p < 0.01, and ***p < 0.001). The details of reagents and primers for the genes were in Supplementary material S2.

## Results

### Data control

After data cleaning, the risk loci are detailed in the Supplementary material, which includes information such as Rsid, Chromosome, Position_GRCh37, Effect allele, Other allele, Eaf (Effect allele frequency), Beta, SE (Standard Error), OR (Odds Ratio), 95% Confidence Interval (95%-CI), and *p*-value. The final selection of phenotypes comprises 416 diseases, with specific details available in Supplementary material S3.

### MR-PheWAS

**Figure 1:**
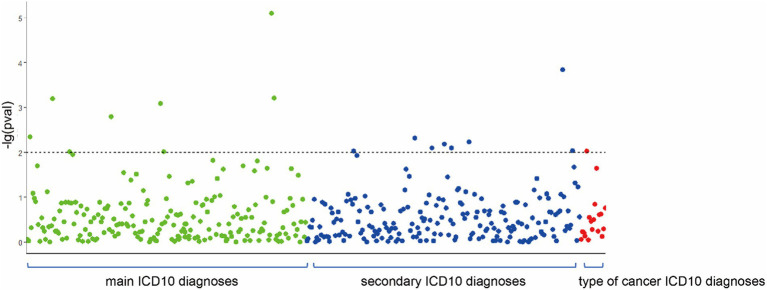
Associations between migraine and 416 diseases.

As depicted in Figure 1, using a threshold of *p* < 0.01, significant associations between migraine and 15 diseases were identified (excluding main ICD10: R51 Headache and main ICD10: G43.9 Migraine, unspecified). Moreover, a causal relationship was established, including: (1) secondary ICD10: Z90.7 Acquired absence of genital organ(s), (2) main ICD10: R69 Unknown and unspecified causes of morbidity, (3) main ICD10: L03.1 Cellulitis of other parts of limb, (4) main ICD10: K25.9 Unspecified as acute or chronic, without hemorrhage or perforation, (5) main ICD10: C44.3 Skin of other and unspecified parts of face, (6) secondary ICD10: K80.2 Calculus of gallbladder without cholecystitis, (7) secondary ICD10: R11 Nausea and vomiting, (8) secondary ICD10: N83.2 Other and unspecified ovarian cysts, (9) secondary ICD10: N32.8 Other specified disorders of the bladder, (10) secondary ICD10: N95.0 Postmenopausal bleeding, (11) secondary ICD10: Z95.1 Presence of aortocoronary bypass graft, (12) Type of cancer: ICD10: C44.3 Skin of other and unspecified parts of face, (13) Diagnoses - secondary ICD10: I25.1 Atherosclerotic heart disease, (14) main ICD10: I21.1 Acute transmural myocardial infarction of the inferior wall, (15) main ICD10: L72.9 Follicular cyst of the skin and subcutaneous tissue, unspecified. Detailed results can be found in the Supplementary material.

For the phenotypes with potential relationships to migraine, we used the MR-Egger method to test for pleiotropy and assess whether it reached balanced pleiotropy. The results indicated significant heterogeneity for secondary ICD10: Z95.1 Presence of aortocoronary bypass graft (*p* = 0.03), and levels of pleiotropy for all phenotypes are presented in Supplementary material S4.

### Single-cell sequencing data analysis

**Figure 2:**
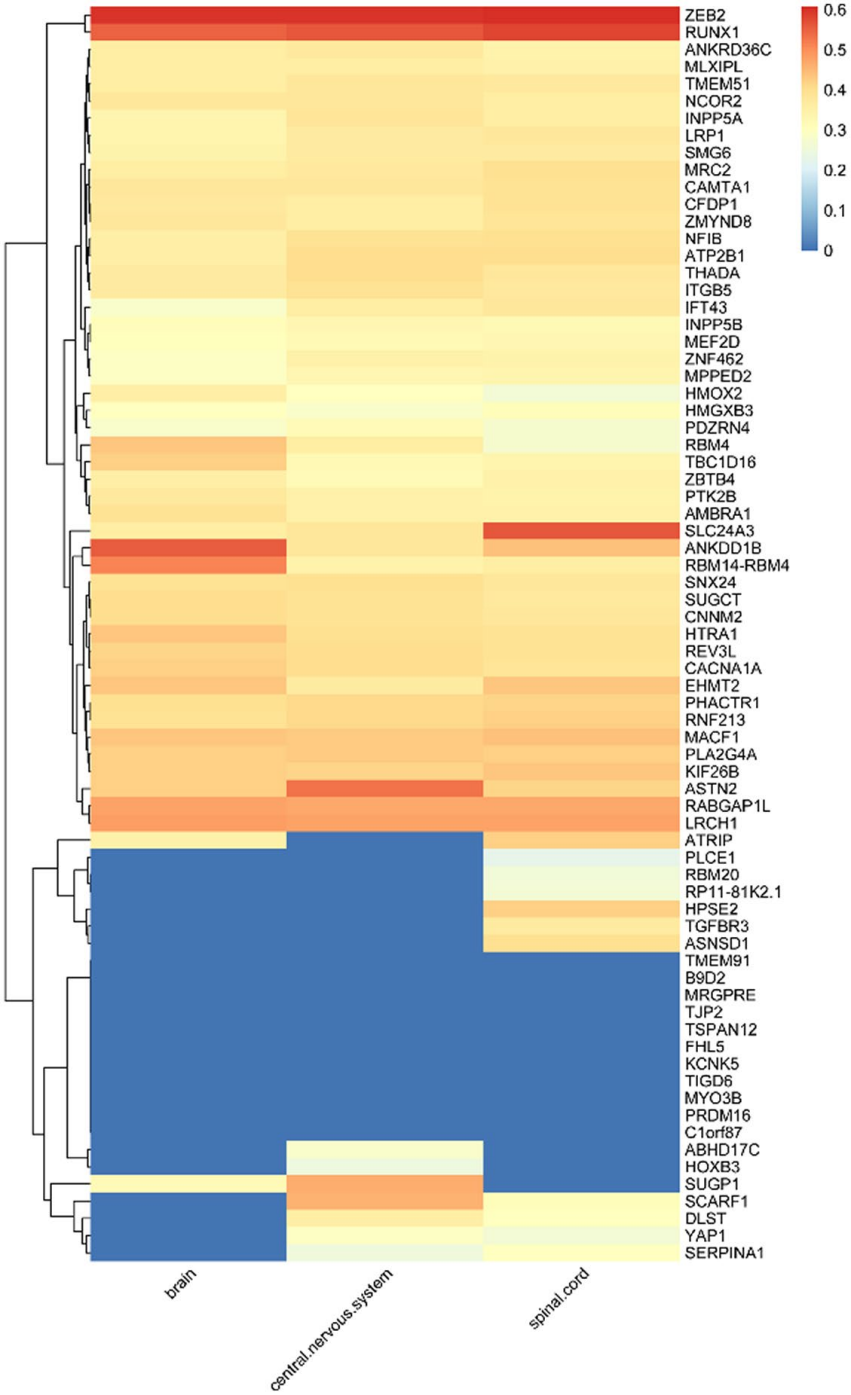
Expression levels of susceptibility genes in microglia.

Of the 123 risk loci associated with migraine, 73 are located within known genes, which we define as susceptibility genes. The expression levels of these 73 susceptibility genes in 16 different cell types within three types of white matter are depicted in Figure 2. Notably, genes such as CAMTA1, MACF1, RABGAP1L, ZEB2, PHACTR1, REV3L, NFIB, ZNF462, ASTN2, ATP2B1, LRCH1, CACNA1A, SLC24A3, RUNX1 exhibit relatively high expression levels across the 16 cell types within these three types of white matter. As illustrated in Supplementary Figure S1, the majority of these 73 genes can be observed to have varying degrees of expression within all three white matter regions. Overall, the expression levels in the brain and spinal cord regions surpass those in the central nervous system.

Given that microglia are considered susceptible cells in migraine, we placed particular emphasis on the investigation of the expression levels of these 73 genes within microglia. As illustrated in Supplementary Figure S2, we observe that ZEB2, RUNX1, SLC24A3, ANKDD1B, RBM14-RBM4, ASTN2, RABGAP1L, and LRCH1 among these susceptibility genes exhibit elevated expression levels in microglia. This observation suggests a potential connection between the expression of these seven susceptibility genes in microglia and their relevance to migraine.

### Verification of the expression level of target genes through qPCR

**Figure 3:**
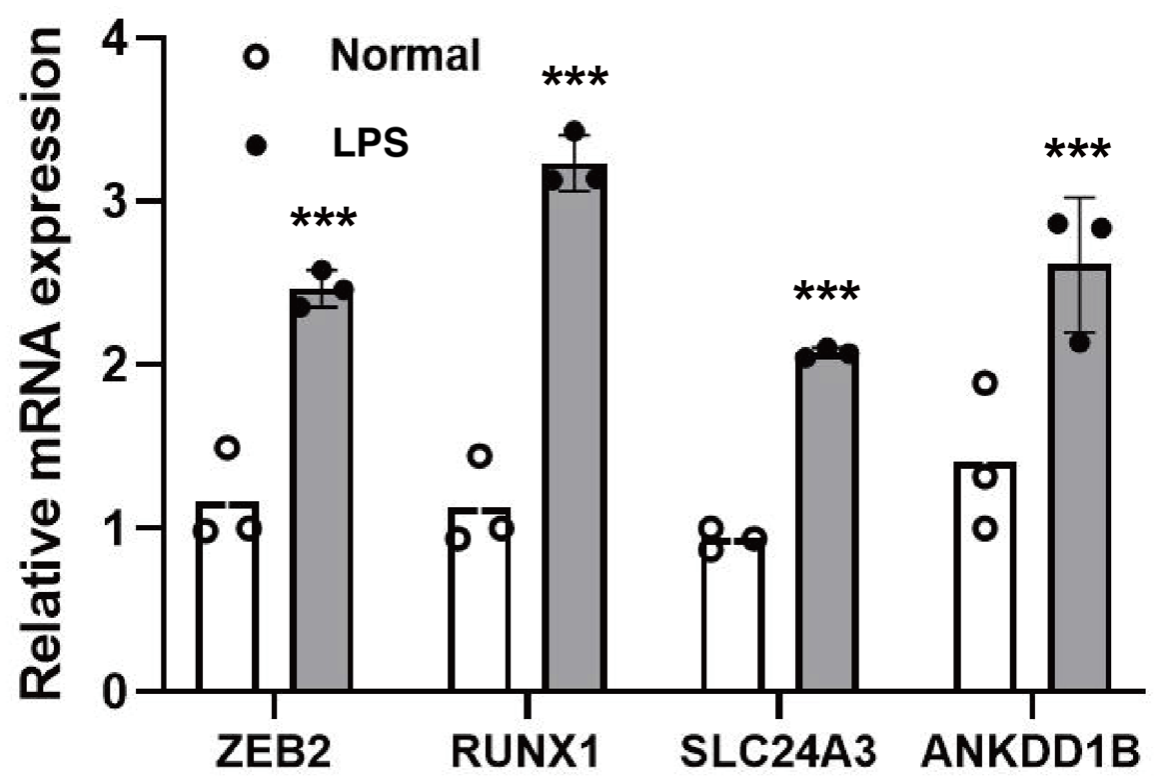
The relative mRNA expression level of target genes. ***p < 0.001.

According to the qPCR results shown in Figure 3, the expression levels of candidate susceptibility genes - ZEB2, RUNX1, SLC24A3, ANKDD1B, RBM14-RBM4, RABGAP1L, and LRCH1—were significantly elevated in microglial cells treated with the inflammatory activator lipopolysaccharide (LPS) compared to untreated control cells. This validation experiment lends strong support to the previous finding from single-cell RNA sequencing analysis, where these four genes exhibited preferential expression in microglia among brain cell types.

## Discussion

Migraine is a complex neurological disorder that is influenced by genetic factors, and understanding the role of genetic factors is crucial to unraveling the mechanisms that underlie the disease. This study integrates cutting-edge genetic and transcriptomic techniques to gain novel insights into the biological basis of migraine. Our PheWAS reveals multiple disease relationships that may reflect shared genetic mechanisms with migraine. In particular, the association with cardiovascular diseases like atherosclerosis is notable given previous clinical links between migraine and stroke risk. The single-cell profiling of migraine risk genes provides clues into specific brain cell types, especially microglia, that may be functionally relevant.

Microglia are vital immune cells in the central nervous system (CNS) that are sensitive to changes in the microenvironment and have a rapid response (20, 21). They are important in the development, maintenance and repair of the CNS and are the dominant regulators of neuroinflammation (22, 23). However, abnormal activation of microglia can lead to migraine headaches (24). For example, IL-17 crosses the blood–brain barrier into the medulla oblongata and, through microglia-mediated neuroinflammation, triggers the activation of the trigeminal nucleus caudalis (TNC), thereby inducing chronic migraine (25).

The results showed that the expression of genes such as ZEB2, RUNX1, SLC24A3 and ANKDD1B was increased in microglia after inflammatory stimulation. This suggests that immune activation could trigger altered gene expression in microglia, contributing to migraine pathogenesis. In the forebrain, ZEB2 has been identified as the first transcription factor (TF) that determines timing of neuro-/gliogenesis, and thus the extent of the different layers of the cortex, in a cell non-autonomous manner (26). ZEB2 was found to play a protective role in astrocyte proliferation and neuronal regeneration after ischemic injury in an *in vivo* MCAO/R rat model (27, 28). RUNX1 is a regulator of hematopoiesis that controls the proliferation of microglia in the postnatal period in response to injury (29). Microglial activation and phenotypic switching play a critical role in neurological disease, and in temporal lobe epilepsy (TLE) mouse model and in BV2 cells, RUNX1 may play a critical role in microglial phenotype through the Notch1 signaling pathway (30). Members of the SLC24 gene family encode K(+)-dependent Na(+)/Ca(2+)exchangers (NCKX), which use both inward Na(+)/outward K(+) gradients to efflux Ca(2+) from cells (31). SLC24A3, also known as Nckx3, is highly expressed in the brain and plays a key role in the intracellular transport of calcium across the cell membrane (32). In addition, during innate immunity and immune responses, Nckx3 plays a critical role. Nckx3 expression is also associated with the p53 and NF-κB pathways during inflammation (33). Excessive microglial activity has been implicated in migraine, so pinpointing specific genetic pathways perturbed in activated microglia is significant. Our results nominate candidate genes and cells for further functional investigation to determine their precise roles. The ANKDD1B gene encodes a protein called ankyrin repeat and death domain containing 1 B (34). In assessing the phenotypic and genetic relationship between migraine and lipoprotein subfractions, ANKDD1B was found to be one of the key genes thought to potentially drive migraine (35). RNA-binding proteins (RBPs) are a family of proteins that play an important role in the regulation of gene expression, either through their canonical function in manipulating the synthesis or processing of RNAs, or through their non-canonical function independent of RNA-binding domains (36). RBM14-RBM4 is a member of this family and plays an important role in neuronal differentiation (37). ASTN2 is located on chromosome 9 and encodes a transmembrane protein that is mainly expressed in the brain (38). Through modulation of synaptic vesicle protein composition, ASTN2 facilitates glial-guided migration during brain development (39). RabGAP1L is a Rab-GTPase activating protein. By binding to the AAMDC protein, RabGAP1L has been shown to activate the PI3K-AKT–mTOR pathway and induce metabolic stress (40). In the brain, microglia are thought to act as neuro-immune sensors, which are sensitive to stress (41). When faced with various environmental stresses and cellular stresses, microglia usually undergo distinctly programmed metabolic changes that lead to diverse function and phenotype (42). LRCH1 encodes a protein with leucine-rich repeats and a structural domain homologous to calpain, which has been shown to be associated with the production of pro-inflammatory cytokines by activated microglia (43).

In our results, single-cell data provides a snapshot of gene expression, so dynamic changes over time remain uncharacterized. Follow-up studies of microglial activation *in vivo* using animal models could better capture the neuroimmunological mechanisms. In summary, integrating genetic associations, transcriptomics, and cell-type specific analyses represents a powerful approach to unraveling migraine etiology. Our findings reveal new links to cardiovascular disease, highlight microglia as a key cellular participant, and nominate gene candidates mediating microglial contributions to migraine pathophysiology. This work helps construct an integrated picture of the polygenic and multifaceted nature of migraine susceptibility.

In this study, we leveraged the valuable resource of UK Biobank data. However, it is essential to acknowledge the potential limitations of our findings concerning the specific population represented in the UK Biobank dataset. The UK Biobank primarily consists of individuals of British descent, and while it offers a rich and diverse dataset, the genetic and environmental factors influencing migraine susceptibility may vary across different populations and ethnicities. Therefore, caution should be exercised when generalizing our results to populations with distinct genetic backgrounds and environmental exposures. To enhance the applicability and robustness of our findings, future research should aim to replicate our findings in more diverse and representative populations, allowing for a more comprehensive understanding of the genetic basis of migraine across different demographic groups.

## Data availability statement

The original contributions presented in the study are included in the article/Supplementary material, further inquiries can be directed to the corresponding author.

## Ethics statement

Ethical approval was not required for the study involving humans in accordance with the local legislation and institutional requirements. Written informed consent to participate in this study was not required from the participants or the participants' legal guardians/next of kin in accordance with the national legislation and the institutional requirements. No animal studies are presented in this manuscript.

## Author contributions

DO: Conceptualization, Data curation, Formal analysis, Writing – original draft, Investigation. CH: Conceptualization, Data curation, Investigation, Methodology, Writing – original draft. HL: Methodology, Project administration, Software, Writing – original draft. WX: Project administration, Software, Validation, Writing – review & editing. CC: Formal analysis, Validation, Visualization, Writing – review & editing. BS: Project administration, Software, Supervision, Writing – review & editing. LG: Funding acquisition, Resources, Supervision, Writing – review & editing.

## Footnotes

^1^ https://ldlink.nih.gov/

^2^ https://data.humancellatlas.org
