# Supplementary material for: Comprehensive analysis of genetic associations and single-cell expression profiles reveals potential links between migraine and multiple diseases: a phenome-wide association study
Source: Front Neurol. 2024 Feb 7;15:1301208. doi: 10.3389/fneur.2024.1301208 (PMC10879407; doi:10.3389/fneur.2024.1301208)
Supplement: Supplementary file 1 [file Presentation_1.pdf]

Supplementary information

1. Reagents

Fetal bovine serum (FBS), Dulbecco modified eagle medium (DMEM) and penicillin-streptomycin were purchased from Gibco Ltd. (New York, USA). Lipopolysaccharides (LPS) was purchased from Sigma-Aldrich (St. Louis, MO, USA). RNA Isolation Kit, First Strand cDNA Synthesis Kit and SYBR Green qPCR Mix were purchased from Sparkjade Biotechnology Co., Ltd. (Shandong, China).

2. The primers for the target genes

Gene	Forward primer	Reverse primer
ZEB2	CAGAAGCCACGATCCAGACC	GGTCAGCAGTTGGGCAAAAG
RUNX1	AACCCAGCATAGTGGTCAGC	CATGGCTGCGGTAGCATTTC
SLC24A3	GCAGGTGAACGACACTCTGA	CAATGGCCAGCGCATAGAAC
ANKDD1B	GAGAGCCAAGAATCAGGATGGA	AGTCATCAGGGTCCCGTGTA
RBM14-RBM4	TGAGGGGTTCAGCCTATCCA	AAATCCCTCAAGGTCAGCCC
ASTN2	CAAGGAGAGTTTCCGCGCTA	CCAAGCTGAAAGTAGACCTGAGA
LRCH1	TCCCCTGCAGTCTCTCCTAC	AGAATGAGGGCTGGGTCTGA
RABGAP1L	CGGAGACGTGAAGAGGTGG	GACTGCTTGGCCTTTTCTCG

3. Supporting Information Figure S1-S2

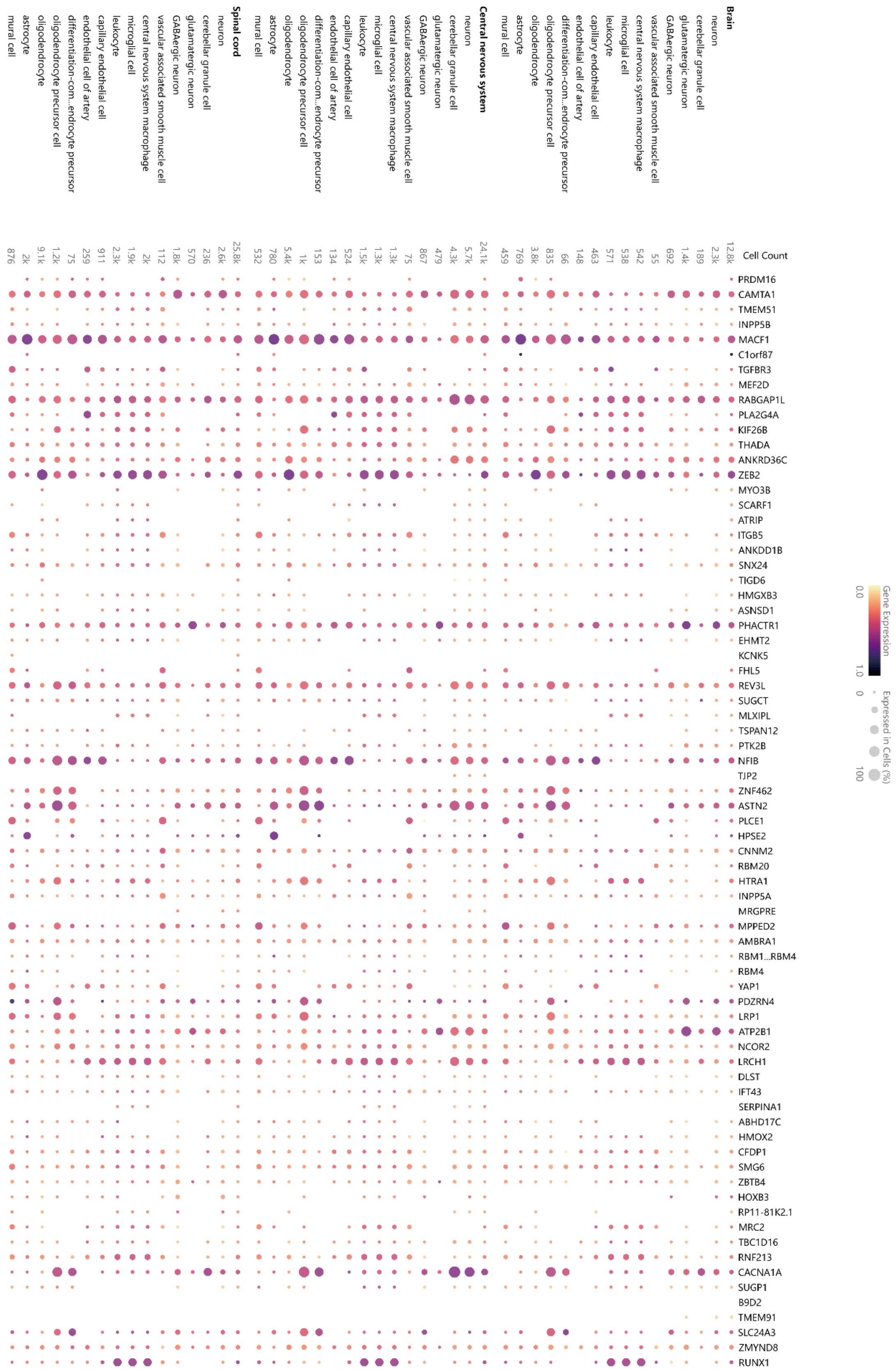

Figure. S1 Expression levels of susceptibility genes in 16 different cell types within three types of

white matter.

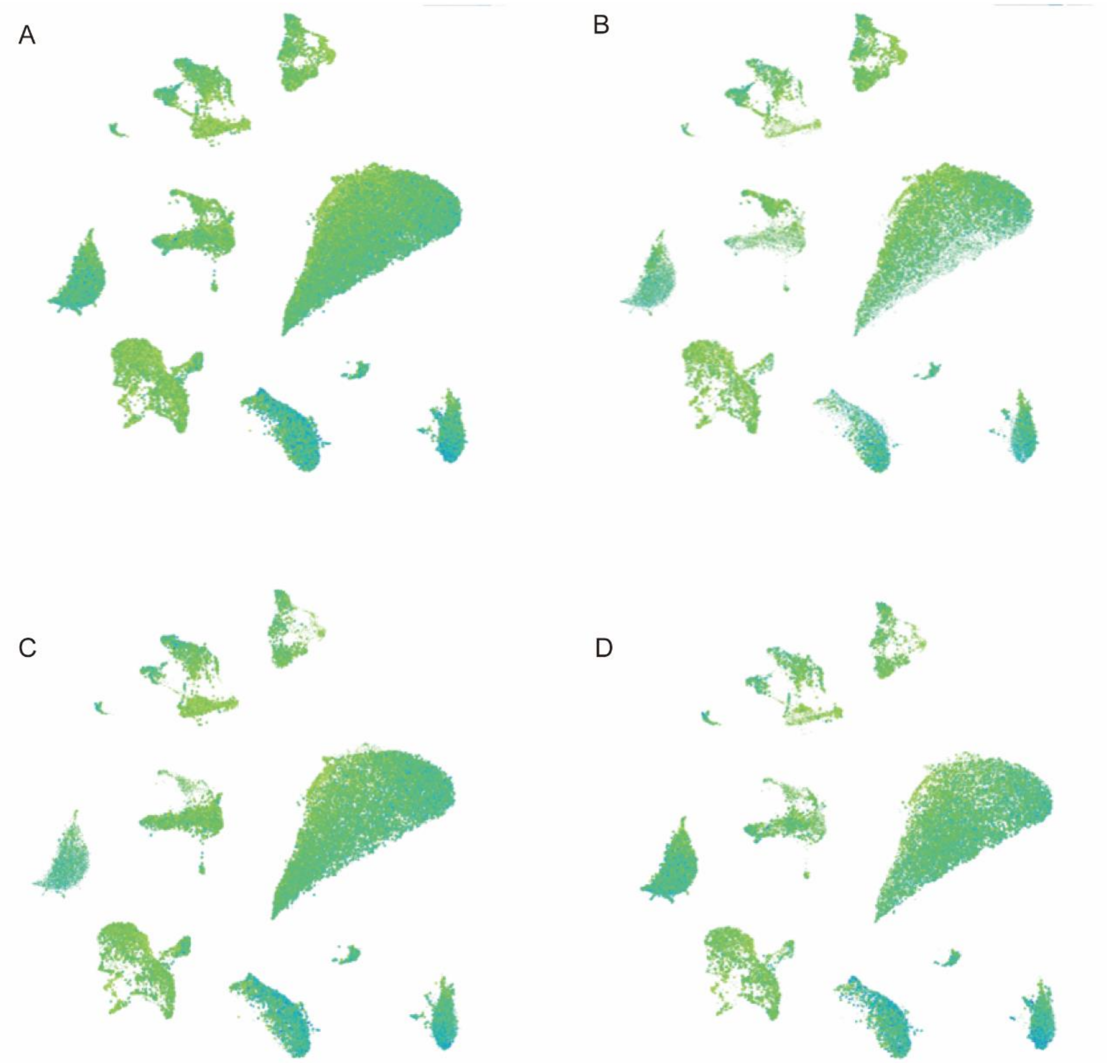

Figure. S2 Expression of gene sets (ZEB2, RUNX1, SLC24A3, ANKDD1B, RBM14-RBM4, ASTN2, RABGAP1L, and LRCH1). (A) Aggregated.(B) Brodmann area 4. (C) Cerebellum. (D)Cervical spinal cord.
